# Signatures of Human NK Cell Development and Terminal Differentiation

**DOI:** 10.3389/fimmu.2013.00499

**Published:** 2013-12-30

**Authors:** Merlin Luetke-Eversloh, Monica Killig, Chiara Romagnani

**Affiliations:** ^1^Innate Immunity, Deutsches Rheuma-Forschungszentrum, Berlin – A Leibniz Institute, Berlin, Germany

**Keywords:** NK cells, ILC, differentiation, IFN-γ, CD62L, CD57, NKG2A, KIR

## Abstract

Natural killer (NK) cells are part of the innate lymphoid cell (ILC) family and represent the main cytotoxic population. NK cells develop from bone marrow common lymphoid progenitors and undergo terminal differentiation in the periphery, where they finally gain their cytotoxic competence as well as the ability to produce IFN-γ in response to engagement of activating receptors. This process has been at least partially elucidated and several markers have been identified to discriminate different NK cell stages and other ILC populations. NK cell terminal differentiation is not only associated with progressive phenotypic changes but also with defined effector signatures. In this essay, we will describe the phenotypic and functional characteristics of the main stages of NK cell development and terminal differentiation and discuss them in light of recent discoveries of novel ILC populations.

## Natural Killer Cells and the Innate Lymphoid Cell Family

Natural Killer (NK) cells are innate lymphocytes with the capability to rapidly kill virus-infected or transformed target cells and to produce type 1 cytokines, such as IFN-γ. Over the past years, other innate lymphoid effectors have been identified, which share defined developmental requirements with NK cells, such as the dependence on the transcriptional repressor inhibitor of DNA binding 2 (Id2), but clearly differ for phenotype and effector properties. The heterogeneity displayed by the different innate lymphocyte subsets closely resembles the one observed among CD4^+^ T helper cells. Thus, a new nomenclature was adapted to collectively define the family, which has been named innate lymphoid cells (ILCs). According to this classification, ILCs have been divided in three main groups: Group 1 ILCs, including NK cells, are defined by the expression of the T-box transcription factors Eomesodermin (*EOMES*) and/or T-bet (*TBX21*) and produce mainly IFN-γ in response to infected or transformed cells; Group 2 ILCs, expressing GATA3, produce IL-13 and IL-5 for the defense against helminthic infections; Group 3 ILCs are characterized by the expression of the orphan nuclear receptor transcription factor RORγt (*RORC*) and include fetal lymphoid tissue-inducer (LTi) cells and adult LTi-like cells, also named ILC3 (1). NK cells likely do not represent the only member of Group 1 ILCs. Indeed, NK cells residing in different organs, such as liver, lung, uterus, or intestine are quite dissimilar among each other. It is still unclear whether this peripheral heterogeneity originates from tissue-specific signals influencing either NK cell *in situ* development or terminal differentiation. Alternatively, tissue resident NK cells might also represent other Group 1 ILC members emerging independently of NK cell precursors. In line with this hypothesis, other potential Group 1 ILC members have been identified recently in the intestine (2, 3). Hence, in the present review we will give an overview on classical human NK cell development and terminal differentiation, and discuss the current knowledge in the frame of this emerging diversity of ILCs.

## Human NK Cell Development

**Figure 1:**
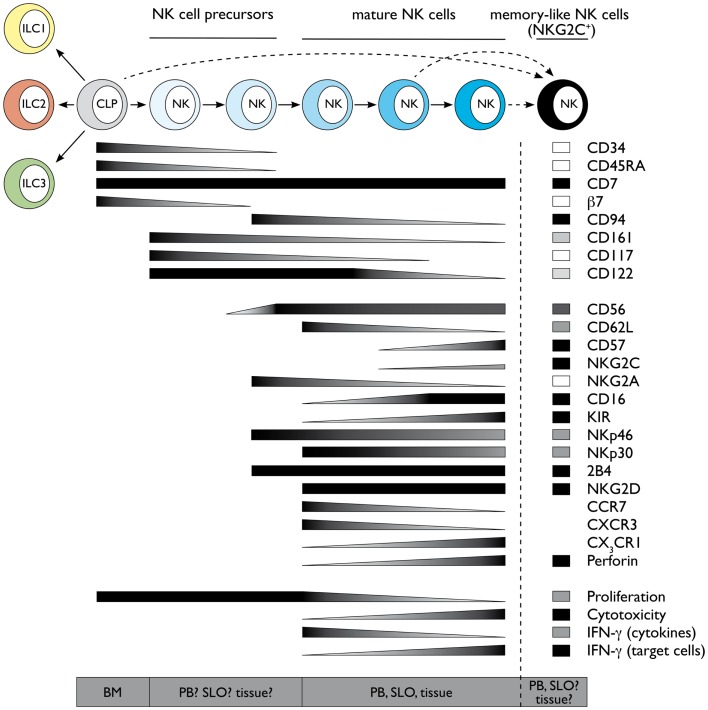
**Schematic representation of human NK cell development and terminal differentiation**. The development of human NK cells from a common lymphoid progenitor over NK cell precursors to terminally differentiated NK cells is depicted from left to right. The acquisition and loss of indicated surface markers and functional properties propose a linear model of human NK cell development and terminal differentiation. Protein expression levels are depicted as black for high expression and white for no expression, gray indicates intermediate levels. Functional properties are indicated correspondingly. Abbreviations: common lymphoid progenitor (CLP), bone marrow (BM), peripheral blood (PB), secondary lymphoid organs (SLO). See also Ref. 59–61.

Natural killer cells develop during fetal life as well as after birth from hematopoietic stem cells (HSCs) through a common lymphoid progenitor (CLP). Although most markers used in the mouse and human systems are different, we will revise the major findings of human NK cell development also in light of murine data. Fetal as well as bone marrow (BM) CLP still have the potential to give rise to B, T, NK, and dendritic cells (DCs). As the development of all mouse ILC group members relies on Id2, it was proposed that a common Id2^+^ ILC progenitor might exist, but still needs to be identified (4). Interestingly, fetal as well as BM HSC can give rise to both, Group 3 ILCs and NK cells in mouse. In particular, expression of the integrin α_4_β_7_ defines a mouse fetal CLP subset with a developmental potential restricted to T, NK, and LTi (Group 3 ILC) cells (5, 6). In line with these data, mouse fetal liver α_4_β_7_^+^ CLP can up-regulate RORγt and differentiate toward LTi cells (7–9). Similarly, after birth, mouse BM precursors expressing integrin α_4_β_7_ and CXCR6 can give rise to Group 3 ILCs as well as to NK cells, although with low efficiency, but have lost B cell as well as T cell differentiation ability, thus potentially represent a common or a mixture of progenitors committed toward Group 3 ILC and NK cell lineage (8). However, it would be relevant to test the differentiation potential of these cells toward Group 2 ILC, which can also be generated from BM CLP (10–12). In humans, a pioneer study from the group of Verneris has clearly shown that total umbilical cord blood-CD34^+^ cells can give rise to both NK cells and Group 3 ILCs (13); however, these populations might originate from fetal CLP, and the identification of a common human ILC progenitor is not yet identified, similar to mouse. In addition to the CLP, mouse NK cell lineage committed progenitors have been initially identified among Lin^−^ CD122^+^ NK1.1^−^ DX5^−^ NK cells (14) and more recently redefined according to the expression of CD27 and CD244 or Id2 (15, 16). Nevertheless, as to date it has not been tested whether these cell populations are also able to differentiate toward Groups 2 or 3 ILCs, it still remains to be formally proven whether they represent or not the earliest NK cell committed precursor. Although the exact phenotype is not yet completely established in humans, CLP are enriched among CD34^+^ CD45RA^+^ CD38^lo^ CD10^±^ CD7^±^ cells. In particular CD34^+^ CD7^+^ CD45RA^+^ and CD34^+^ CD10^+^ CD45RA^+^ cells are preferentially biased to develop into T/NK and B cells, respectively (17–20). CLP-like cells with NK cell commitment potential and expressing β_7_ integrin, similar to mice, have also been described in human peripheral blood (PB) and shown to be enriched in extramedullary sites, such as secondary lymphoid organs (SLOs) (21–23), which were therefore proposed as putative sites of human NK cell development. According to this concept, human CLP-like CD34^+^ β_7_ integrin^+^ CD45RA^+^ (c-Kit^−^ CD94^−^) cells identified in SLO still display T cell and DC potential and have been termed “stage I” NK cells (23). Human NK cell differentiation in SLO would then proceed through “stage II” (CD34^+^ CD45RA^+^ c-Kit^+^ CD94^−^) and “stage III” (CD34^−^ c-Kit^+^ CD94^−^) NK cells, which would finally give rise to “stage IV” NK cells, also defined as CD56^bright^ CD16^neg/lo^ NK cells (23). More recently, it has been shown that most putative “stage III” human NK cells (CD34^−^ c-Kit^+^ CD94^−^) isolated *ex vivo* in SLO are actually mature ILC3 (24, 25). As mature human ILC3 mostly coexpress c-Kit and CD127, it was proposed that among CD34^−^ c-Kit^+^ cells only a minor fraction, characterized by the lack of CD127 might represent “stage III” human NK cells (25). Thus, due to the complex overlapping of markers between human NK cells and ILC3, a more detailed analysis of *in vitro* differentiation of “stage III” and “stage II” NK cell precursors toward different ILC subsets would be of great help. Nevertheless, these studies have contributed crucially to support the idea that extramedullary compartments might indeed represent important sites of NK cell development (22, 23). The unique milieu available in different tissues might influence *in situ* differentiation of NK cells and could actually explain the large phenotypic and functional heterogeneity found among NK cells derived from different tissues, such as liver, lung, or uterus. NK cell precursors might migrate very early to the tissues and develop *in situ* influenced by tissue-specific signals, such as cytokines, stromal or epithelial cells, and environmental cues. However, we cannot exclude that this large heterogeneity might actually rather reflect potentially novel Group 1 ILC subsets, displaying NK-like phenotype. Along this line, extramedullary compartments might represent preferential developmental sites not only for most Group 1 but also for Group 3 ILCs. It has been shown that precursors of murine Group 3 ILCs do not up-regulate RORγt expression in the BM and might migrate very early to the periphery, especially to the intestinal lamina propria (LP), for their development/terminal differentiation (8, 26). However, it still remains an open question at which developmental stage (CLP? common ILC, Group 1 ILC, or NK cell precursor?) NK cells migrate to peripheral tissues to undergo further differentiation. Figure 1 illustrates markers associated with early NK cell developmental stages derived from human CLP.

## Human NK Cell Terminal Differentiation and Related Effector Functions

Despite the presence of NK cells or NK cell-like populations in different organs, most of our knowledge on NK cell biology originates from mouse splenic and human PB-derived NK cells. Even within the same compartment, NK cells are heterogeneous, and one possible interpretation of this observation is that this diversity might reflect a process of terminal differentiation occurring in the periphery. Along this line, different markers have been used in humans and mouse to describe the stages of final NK cell maturation. In mouse, several authors have contributed to delineate a model in which splenic NK1.1^+^ NK cells can be mainly dissected according to CD27 and CD11b, which enable to foresee the following maturation progression: CD27^−^ CD11b^−^ → CD27^+^ CD11b^−^ → CD27^+^ CD11b^+^ → CD27^−^CD11b^+^ NK cells (27–29). Conversely, most of the data concerning this process in humans relies on the analysis of circulating PB-derived NK cells. In this compartment, two main NK subsets were originally described on the basis of the differential expression of CD56, which has no homolog in mice and were named CD56^bright^ and CD56^dim^ NK cells (30, 31). CD56^bright^ and CD56^dim^ NK cells differ in phenotype, functional capabilities, and preferential locations. CD56^bright^ NK cells are only a minority of PB-NK cells, whereas they represent the majority of NK cells in SLO. CD56^bright^ NK cells have been described as preferentially CD62L^−/lo^ CD94/NKG2A^+^ CD62L^hi^ CD57^−^ KIR^−^ and commonly termed as the immunomodulatory subset, because of their potential to produce high levels of cytokines like IFN-γ and TNF. In contrast, they express only very low amounts of cytolytic granules and display poor cytotoxic capability. This feature is a characteristic of CD56^dim^ NK cells, which express high amounts of perforin and granzymes and are able to kill target cells like virus-infected and transformed cells. However, the idea that CD56^bright^ are the cytokine producers whereas CD56^dim^ NK cells represent the cytotoxic subset, might be actually misleading as both NK cell subsets can produce large amounts of IFN-γ. CD56^bright^ NK cells produce high amounts of IFN-γ and extensively proliferate in response to DC-derived cytokines like IL-2, IL-15, IL-12, and IL-18 (31), but are unable to produce IFN-γ in response to target cell recognition (32, 33). Conversely, CD56^dim^ NK cells are less efficient in proliferating and producing IFN-γ in response to cytokines (31), but become the main IFN-γ producers after target cell encounter (32, 33). A detailed analysis of CD56^dim^ functional properties and phenotype has actually revealed a consistent heterogeneity also among CD56^dim^ NK cells. When CD56^dim^ NK cells were dissected according to the expression of either CD62L, CD94/NKG2A, or lack of CD57, a subset of NK cells with intermediate phenotype and properties between CD56^bright^ (CD94/NKG2A^hi^ CD62L^hi^ CD57^−^) and CD56^dim^ CD94/NKG2A^–^, CD62L^–^, or CD57^+^ cells could be identified (33–39). These data were also confirmed at global transcriptome level (33). CD56^dim^ NK cells displaying this intermediate signature (CD94/NKG2A^+^, CD62L^+^, or CD57^−^) combine the ability to produce IFN-γ and proliferate in response to cytokines, characteristic for CD56^bright^ NK cells, with the capacity to kill and produce IFN-γ upon engagement of activating receptors (actRs), typical of CD56^dim^ NK cells. The developmental relationship between CD56^bright^ and the different CD56^dim^ NK cell subsets has been controversially discussed, but several studies support the concept that CD56^bright^ cells might represent the progenitors of CD56^dim^ NK cells. CD56^bright^ NK cells display longer telomeres compared to CD56^dim^ NK cells and can acquire the expression of CD16 and killer Ig-like receptors (KIR), as well as of other markers, *in vitro* as well as *in vivo* after transfer into immunodeficient mice (33, 36). Further observations support a model of linear maturation from more immature CD56^bright^ toward terminally differentiated CD56^dim^ (CD94/NKG2A^–^, CD62L^–^, or CD57^+^) NK cells passing through CD56^dim^ NK cell subsets displaying intermediate signatures (CD94/NKG2A^+^, CD62L^+^, or CD57^–^). As previously mentioned, this phenotypic sequence is also associated with progressive loss of responsiveness to cytokines and gradual acquisition of responsiveness to actR engagement (33–35) (Luetke-Eversloh and Romagnani, unpublished data). The capability of the more immature CD56^bright^ and CD56^dim^ NK cell subsets to respond to cytokine stimulation strictly correlates to the expression of CD94, CD62L, or CD57 and is reflected by higher expression of cytokine receptors and STAT4 activation (33, 37, 39, 40). Conversely, the ability of the CD56^dim^ subsets to kill and produce IFN-γ in response to actR mainly correlates to the expression of self MHC class I binding inhibitory receptors, in humans belonging to the KIR family or CD94/NKG2A (41–44). This phenomenon, termed education or licensing, was originally described in mice for NK cells expressing self MHC class I specific inhibitory Ly49 receptors or CD94/NKG2A (41–43). According to this concept, during their differentiation or the course of immune responses, NK cells are licensed by self MHC class I molecules through engagement of their inhibitory receptors and this interaction results in NK cell functional competence in response to actR. Thus, licensed NK cells are not only functionally competent but also tolerant because they display at least one inhibitory receptor for self MHC. Conversely, NK cells which do not have a self MHC-specific inhibitory receptor are less functional and therefore still tolerant toward self (41, 45–47). The molecular mechanisms underlying this process have been only partially clarified. In mice, NK cell education seems to require functional immunoreceptor tyrosine-based inhibitory motifs (ITIMs) of self MHC class I binding Ly49 receptors, but to be independent of SHP-1 signaling (41–43). In addition, educated mouse NK cells have an altered membrane distribution of actR, which is instrumental to functional competence in response to actR engagement (41, 45–47). Although further research is needed to clarify the site, the stimuli, and the time of NK cell education, it is now clear that this process is not an on-off switch occurring during BM development, but rather a fine tuning determined by the number and the affinity of MHC-inhibitory receptor interactions (41, 45–48). However, the stage of NK cell terminal differentiation might also contribute to finely tune functional competence in response to actR engagement. Terminally differentiated NK cell subsets are enriched in cells expressing more than one inhibitory receptor (39), and thus in educated cells, suggesting that differentiation and education might not be entirely uncoupled processes. In Figure 1, we summarized several markers defining the phenotype and functions of the different NK cell subsets.

In contrast to individual markers, the combination of expression of CD16, KIR, CD57, CD62L, CD94/NKG2A does not identify three main populations but results in a complex number of intermediate, terminally differentiated, educated, and non-educated NK cell subsets. Although this implies even a larger heterogeneity of PB-NK cells, it does not exclude that NK cell terminal differentiation still proceeds following a linear progression. In support of this model, we have observed that these markers are not acquired or lost synchronously in time during reconstitution from HSC during transplantation and this might contribute to explain NK cell peripheral variety (M.K. and C.R., unpublished data). Moreover, diverse signals, such as homeostatic and pro-inflammatory cytokines or engagement of defined activating or inhibitory receptors at steady state or during infections, might influence NK cell terminal differentiation and/or education and determine the quality and the intensity of this process. One clear example of how environmental stimuli can dramatically poise human NK cell final maturation is cytomegalovirus (CMV) infection, which can globally accelerate NK cell terminal differentiation (49). CMV infection induces the expansion and persistence of a unique NK cell subset, expressing the actR NKG2C and being preferentially positive for CD57 and self MHC class I binding KIR (50–52). Although NKG2C^+^ NK cells undergoing expansion during CMV infection tend to resemble phenotypically and functionally terminally differentiated NK cells, it has been also suggested that they might represent the human correspondent of the previously described Ly49H^+^ memory-like murine NK cells expanding and persisting after CMV infection (53, 54) (Figure 1). Interestingly, expansion of NKG2C^+^ NK cells occurs also in CMV-seropositive patients with chronic hepatitis B or C virus or during hantavirus infection (55, 56). Further experiments are required to understand their origin and developmental relationship with other PB-NK cell subsets.

Interestingly, different stages defined according to CD16, NKG2A, and KIR can also be identified among fetal NK cells, suggesting that similar forces likely govern the process of differentiation before and after birth. However, even in fetal lung where NK cells are strongly enriched in more mature KIR^+^ cells, CD57 expression is very low and only NKG2A, but not self MHC specific KIR, mediate NK cell education, possibly suggesting that fetal tissue milieu influence NK cell differentiation and functional properties (57).

Despite of all these suggestive evidences, it is however difficult to completely exclude that human NK cell peripheral heterogeneity might not rather represent developmentally unrelated members of the Group 1 ILC family. CD56^bright^ NK cells uniquely express CD127, CD117, and GATA3, which are hallmarks of other ILC subsets and have been also proposed to correspond to mouse thymus-derived NK cells (58). Further investigation employing different experimental approaches might help us to revisit this issue and elicit some surprises.

“It’s a rare occasion when your plans and expectations come down just exactly how you planned them. Who can say, anyway? Time will tell” (“Time will tell,” Tower of Power).

## Conflict of Interest Statement

The authors declare that the research was conducted in the absence of any commercial or financial relationships that could be construed as a potential conflict of interest.
